# Metabolism and antioxidant defense in the larval chironomid *Tanytarsus minutipalpus*: adjustments to diel variations in the extreme conditions of Lake Magadi

**DOI:** 10.1242/bio.021139

**Published:** 2016-11-28

**Authors:** Lucas F. Bianchini, Chris M. Wood, Harold L. Bergman, Ora E. Johannsson, Pierre Laurent, Claudine Chevalier, Mosiany L. Kisipan, Geraldine D. Kavembe, Michael B. Papah, Kevin V. Brix, Gudrun De Boeck, John N. Maina, Rodi O. Ojoo, Adalto Bianchini

**Affiliations:** 1 Programa de Pós-Graduação em Ciências Fisiológicas: Fisiologia Animal Comparada, Instituto de Ciências Biológicas, Universidade Federal do Rio Grande, Avenida Itália km 8, Rio Grande, RS 96203-900, Brazil; 2 Department of Biology, McMaster University, Hamilton, Ontario, CanadaL8S 4K1; 3 Department of Zoology, University of British Columbia, Vancouver, British Columbia, CanadaV6T 1Z4; 4 Department of Zoology and Physiology, University of Wyoming, Laramie, WY 82071, USA; 5 Department of Veterinary Anatomy and Physiology, Faculty of Veterinary Medicine and Surgery, Egerton University, P.O. Box 536 - 20115, Egerton, Kenya; 6 Department of Biology, South Eastern Kenya University, Kitui 170-90200, Kenya; 7 Department of Veterinary Anatomy and Physiology, University of Nairobi, P.O. Box 30197, Nairobi 00100, Kenya; 8 EcoTox, Miami, FL 33155, USA; 9 SPHERE, Department of Biology, University of Antwerp, Antwerp 2020, Belgium; 10 Department of Zoology, University of Johannesburg, Johannesburg 2006, South Africa

**Keywords:** Adjustment, Glutathione, Insect, Reactive oxygen species, Oxidative stress, Urea

## Abstract

Insect larvae are reported to be a major component of the simple but highly productive trophic web found in Lake Magadi (Kenya, Africa), which is considered to be one of the most extreme aquatic environments on Earth. Previous studies show that fish must display biochemical and physiological adjustments to thrive under the extreme conditions of the lake. However, information for invertebrates is lacking. In the present study, the occurrence of the larval chironomid *Tanytarsus minutipalpus* is reported in Lake Magadi for the first time. Additionally, changes in larval metabolism and antioxidant defense correlated with diel variations in the extremely hostile environmental conditions of the lake are described. Wide variations in water temperature (20.2-29.3°C) and dissolved oxygen content (3.2-18.6 mg O_2_ l^−1^) were observed at different times of day, without significant change in water pH (10.0±0.03). Temperature and dissolved oxygen were higher at 13:00 h (29.3±0.4°C and 18.6±1.0 mg O_2_ l^−1^) and 19:00 h (29.3±0.8°C and 16.2±1.6 mg O_2_ l^−1^) and lower at 01:00 h (21.1±0.1°C and 10.7±0.03 mg O_2_ l^−1^) and 07:00 h (20.2±0.4°C and 3.2±0.7 mg O_2_ l^−1^). Significant and parallel increases in parameters related to metabolism (cholinesterase, glucose, cholesterol, urea, creatinine and hemoglobin) and the antioxidant system (SOD, GPx, GR, GSH and GSSG) were observed in larvae collected at 13:00 h. In contrast, no significant changes were observed in pro-oxidants (ROS and NO), TOSC and oxidative damage parameters (LPO and DNA damage). Therefore, the observed increases in temperature and dissolved O_2_ content in Lake Magadi were associated with changes in the antioxidant system of *T. minutipalpus* larvae. Adjustments performed by the chironomid larvae were efficient in maintaining body homeostasis, as well as protecting biomolecules against oxidative damage, so that oxidative stress did not occur. GSH-GSSG and GPx-GR systems appeared to play an essential role in the adjustments displayed by the chironomid larvae during the diel changes in the extreme conditions of Lake Magadi.

## Abstract (precis)

**Summary:** Insect larvae display adjustments in metabolism and oxidative status to overcome the diel variations in the extreme and harsh physicochemical conditions of Lake Magadi, a saline and alkaline lake in Kenya.

## INTRODUCTION

Lake Magadi in southern Kenya (East Africa) represents the most saline water body of the African Rift Valley (Kodikara et al., 2012). Additionally, waters coming from the northwestern and southeastern thermal springs in the lake can reach up to 86°C (Jones et al., 1977). Physicochemical analyses of Lake Magadi waters indicate marked diel changes in temperature (20–43°C) and dissolved oxygen content (3–18 mg O_2_ l^−1^). Indeed, this lake is considered to be one of the most extreme aquatic environments on Earth (pH ∼10, carbonate alkalinity ∼380 mEq l^−1^; specific density: 1.015; osmolality: ∼600 mosmol l^−1^; and high incidence of ultraviolet light) (Reite et al., 1974; Johansen et al., 1975; Narahara et al., 1996; Wilson et al., 2004; Johannsson et al., 2014).

The tolerance of aerobic aquatic animals to extreme environmental conditions, especially temperature and dissolved oxygen content, is dependent on morphological, biochemical, physiological and behavioral adaptations (Hermes-Lima, 2004; Wilson et al., 2004; Régnière et al., 2012; Johannsson et al., 2014). Therefore, animals inhabiting Lake Magadi must have evolved adaptive processes to colonize and thrive under the extreme conditions of the lake. For example, Randall et al. (1989) and Wood et al. (1989) showed that *Alcolapia grahami* (formerly *Oreochromis alcalicus grahami*), a tilapia endemic to Lake Magadi, excretes all nitrogenous waste as urea rather than ammonia, thus being different from most fish. Although several studies have focused on the responses of *A. grahami* to the extreme environmental conditions of Lake Magadi (for example, Reite et al., 1974; Johansen et al., 1975; Randall et al., 1989; Wood et al., 1989, 1994; Narahara et al., 1996; Wilson et al., 2004; Wood et al., 2012, 2013; Johannsson et al., 2014), there have been no similar studies on invertebrates of this lake. A simple but highly productive trophic web exists in the extreme conditions of Lake Magadi. This food web is composed of the cyanobacteria *Arthrospira* sp., copepods, insect larvae, the tilapia *A. grahami*, and a variety of piscivorous birds (e.g. egrets, herons, terns, gulls and pelicans) and flamingos (Coe, 1966; Javor, 1989; Johannsson et al., 2014). Although the presence of insect larvae has been reported previously in the lake, they were never taxonomically identified. Therefore, during expeditions to Lake Magadi in 2010 and 2013, chironomid larvae were collected, identified at the species level, and analyzed for possible biochemical and physiological adjustments correlated with diel variations in the harsh and extreme conditions of the lake.

Among aquatic insects, those belonging to the family Chironomidae show a wide distribution around the world, being present in all zoogeographical regions (Trivinho-Strixino, 2010). They can be found in almost all freshwater environments (lotic and lentic systems), thermal springs, volcanic lakes, melting waters, brackish waters, intertidal zones and even in oil wells (Pinder and Reiss, 1983); and they can be saprophytes, herbivores, detritivores or predators (Merritt and Cummins, 1984; Ward, 1992; Trivinho-Strixino and Strixino, 1995). Just like all other insects belonging to the order Diptera, chironomids undergo a complete metamorphosis during their life cycle. Juvenile stages and adults show morphological, physiological, behavioral and ecological differences (Chapman, 1998; Jurenka, 2015). Larvae of most species are aquatic, with some occurring under organic matter undergoing decomposition, barks of plants or humid soils; adults generally have a very short lifespan and do not feed, with few exceptions (Rasmussen, 1996; Jurenka, 2015).

In the context of the present study, it is worth noting that water temperature and dissolved oxygen content have a great influence on the metabolism of aquatic insects, including chironomids (Rasmussen, 1996; Chapman, 1998; Corbi and Trivinho-Strixino, 2006; Jurenka, 2015). In turn, the oxygen needed to fulfill the metabolic requirements of chironomids is stored by the hemoglobin present in tissues (Lankester, 1867; Walshe, 1947; Panis et al., 1995; Rasmussen, 1996); therefore, changes in insect metabolism induced by increasing water temperature or changes in dissolved oxygen content can be evaluated using alternative indicators such as hemoglobin and its metabolite bilirubin. Similarly, changes in ventilation and locomotor behavior in insects can be evaluated through cholinesterase activity (Jensen et al., 1997; Azevedo-Pereira et al., 2011).

In most cases, increased environmental temperature is paralleled by an enhanced metabolism in insects, which could be reflected by changes in the amount of free and stored energy substrates such as carbohydrates, lipids and proteins, as well as their metabolites (Chapman, 1998; Jurenka, 2015). However, significant changes have generally been observed more often in carbohydrate and lipid stores than in protein levels. Creatine and creatinine are found in the circulating fluid, fat body and excreta of insects (Lazar and Mohamed, 1988a,b). Therefore, these compounds can be used as alternative indicators of changes in protein metabolism in insects.

Extreme conditions and marked diel variations in temperature and dissolved oxygen content have been reported in Lake Magadi for decades (Reite et al., 1974; Johansen et al., 1975; Narahara et al., 1996; Wilson et al., 2004). Recently, we demonstrated that Lake Magadi waters also exhibit exceptional levels of reactive oxygen species (ROS) that can reach up to 8.10 μM H_2_O_2_, among the highest ever reported for any water body (Johannsson et al., 2014). Probably, the marked increases in daytime water temperature and dissolved oxygen content observed in Lake Magadi waters (Reite et al., 1974; Johansen et al., 1975; Narahara et al., 1996; Wilson et al., 2004) could lead to excessive oxygen consumption followed by an enhanced generation of ROS, which could induce oxidative stress. Indeed, a worse scenario may occur when this condition is combined with the high levels of ROS found in Lake Magadi waters (Johannsson et al., 2014).

In light of the above, we predicted that the chironomid larvae of Lake Magadi would display biochemical and physiological adjustments to deal with the extreme environmental conditions. In particular, we hypothesized that when chironomid larvae were exposed to natural changes in environmental conditions, especially temperature and dissolved oxygen content, they would show increased metabolism and that defense would be provided by the antioxidant (enzymatic and non enzymatic) system against ROS. In turn, this would avoid oxidative stress and consequent damage to biomolecules. Therefore, the main goal of the present study was to evaluate changes in metabolism and oxidative status in chironomid larvae found in Lake Magadi during the natural diel changes observed in the extreme environmental conditions of the lake. To achieve our goal, major water physicochemical parameters of Lake Magadi were measured and chironomid larvae were collected at five time points over a continuous 24-h period. Whole larvae were analyzed for 22 parameters related to metabolism and oxidative status using spectrophotometric methods. Biochemical and physiological parameters evaluated included energy substrates, metabolites, respiratory pigments, enzyme activities, oxidants, antioxidants, and oxidative damage to biomolecules.

## RESULTS

The larval chironomids used in the present study were taxonomically identified as *Tanytarsus minutipalpus* (Ekrem and Harrison).

**Fig. 1.**
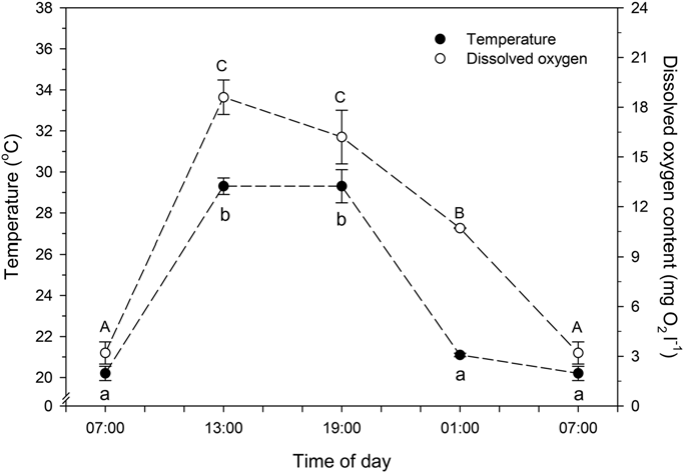
**Diel variations in water temperature and dissolved oxygen content in Flamingo Lagoon in Lake Magadi (Magadi Township, Kajiado County, Rift Valley, Kenya, Africa).** Measurements were performed in July 2010 and 2013. Data are mean±s.e.m. (*n*=6). Different letters indicate significantly different mean values among times of day for each environmental parameter (one-way analysis of variance followed by Tukey's test; *P*<0.05).

Water physicochemical parameters measured in Flamingo Lagoon (Lake Magadi) during late July, the coolest time of the year, are shown in Fig. 1. Wide variations in water temperature (20.2–29.3°C) and dissolved oxygen content (3.2–18.6 mg O_2_ l^−1^) were observed at the different sampling times. Higher mean water temperature and dissolved oxygen content were observed at 13:00 h and 19:00 h, whereas lowest values occurred at 01:00 h and 07:00 h (Fig. 1). In contrast, there was no significant diel variation of water pH and therefore a mean value was calculated for all sampling times (pH 10.0±0.03).

**Fig. 2.**
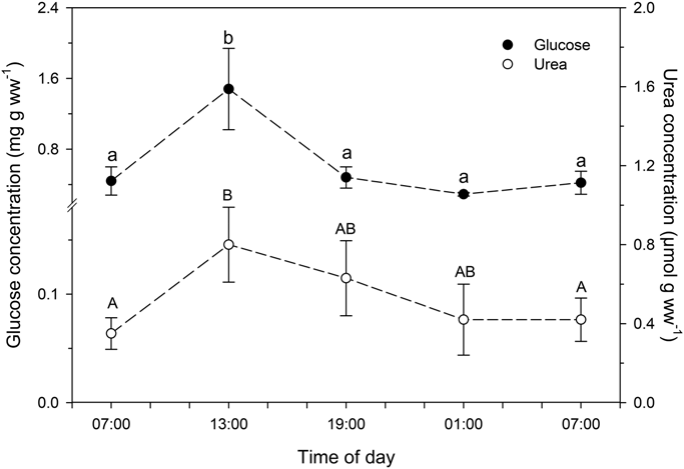
**Whole-body glucose and urea concentrations in larvae of the chironomid *Tanytarsus minutipalpus* collected from Flamingo Lagoon in Lake Magadi at different times of day.** Data are mean±s.e.m. (*n*=6 pools; 10 larvae per pool). Different letters indicate significantly different mean values among chironomids sampled at different times of day for each parameter analyzed (one-way analysis of variance followed by Tukey's test; *P*<0.05).

**Fig. 3.**
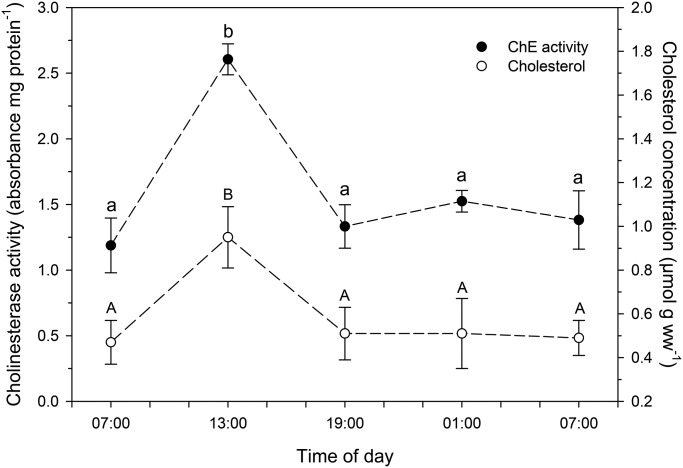
**Whole-body cholinesterase (ChE) activity and cholesterol concentration in larvae of the chironomid *Tanytarsus minutipalpus* collected from Flamingo Lagoon in Lake Magadi at different times of day.** Data are mean±s.e.m. (*n*=6 pools; 10 larvae per pool). Different letters indicate significantly different mean values among chironomids sampled at different times of day for each parameter analyzed (one-way analysis of variance followed by Tukey's test; *P*<0.05).

**Fig. 4.**
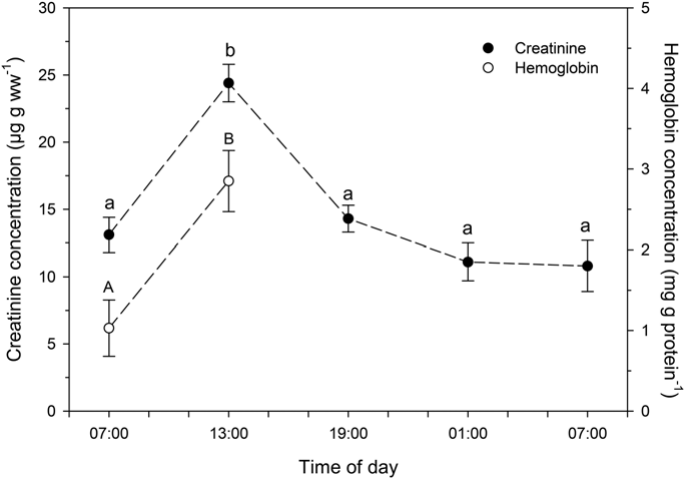
**Whole-body creatinine and hemoglobin concentrations in larvae of the chironomid *Tanytarsus minutipalpus* collected from Flamingo Lagoon in Lake Magadi at different times of day.** Data are mean±s.e.m. (*n*=6 pools; 10 larvae per pool). Different letters indicate significantly different mean values among chironomids sampled at different times of day for each parameter analyzed (one-way analysis of variance followed by Tukey's test; *P*<0.05).

Data for several indicators of chironomid metabolic activity are shown in Table 1. There were no significant changes in whole-body concentrations of triglycerides, proteins, bilirubin and mucoproteins in larvae collected at the different times of day. Also, no significant diel changes were observed in the concentrations of pro-oxidants (ROS and NO), as well as in the total antioxidant capacity (TOSC). Furthermore, no significant variations were observed in LPO and DNA damage levels in larvae collected at the different times of day. However, whole-body glucose (Fig. 2), cholinesterase activity, cholesterol (Fig. 3), creatinine and hemoglobin (Fig. 4) concentrations were significantly higher in chironomid larvae collected at 13:00 h than in those collected at the other times of day (07:00 h and 19:00 h of the first day, and 01:00 h and 07:00 h of the next day). Whole-body urea concentration was higher in chironomid larvae collected at 13:00 h than in those collected in the morning (07:00 h of the first day and 07:00 h of the following day) (Fig. 2).

**Table 1.**
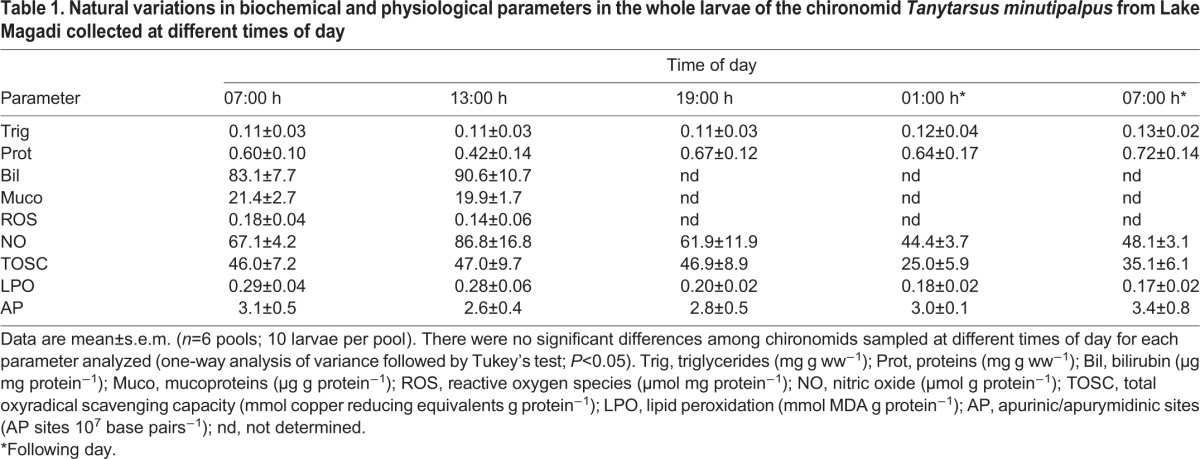
**Natural variations in biochemical and physiological parameters in the whole larvae of the chironomid *Tanytarsus minutipalpus* from Lake Magadi collected at different times of day**

**Fig. 5.**
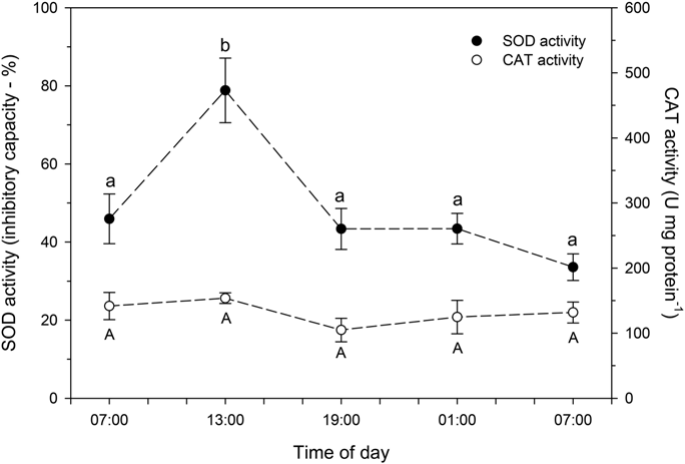
**Whole-body superoxide dismutase (SOD) and catalase (CAT) activity in larvae of the chironomid *Tanytarsus minutipalpus* collected from Flamingo Lagoon in Lake Magadi at different times of day.** Data are mean±s.e.m. (*n*=6 pools; 10 larvae per pool). Different letters indicate significantly different mean values among chironomids sampled at different times of day for each parameter analyzed (one-way analysis of variance followed by Tukey's test; *P*<0.05).

**Fig. 6.**
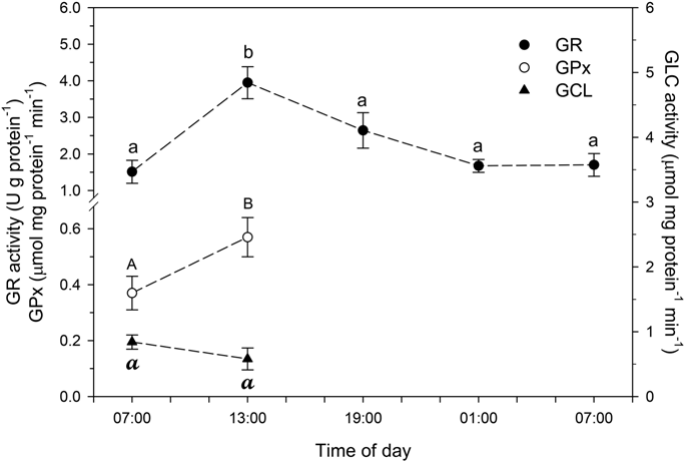
**Whole-body glutathione reductase (GR), glutathione peroxidase (GPx) and glutamate-cysteine ligase (GCL) activity in larvae of the chironomid *Tanytarsus minutipalpus* collected from Flamingo Lagoon in Lake Magadi at different times of day.** Data are mean±s.e.m. (*n*=6 pools; 10 larvae per pool). Different letters indicate significantly different mean values among chironomids sampled at different times of day for each parameter analyzed (one-way analysis of variance followed by Tukey's test; *P*<0.05).

**Fig. 7.**
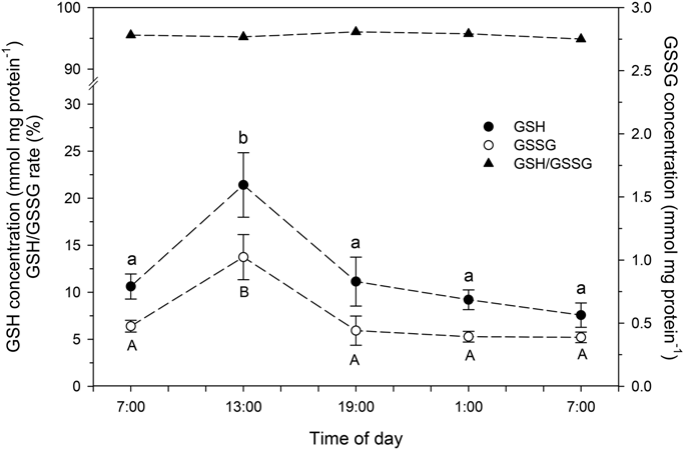
**Whole-body reduced glutathione (GSH), oxidized glutathione (GSSG) and GSH/GSSG concentration ratio in larvae of the chironomid *Tanytarsus minutipalpus* collected from Flamingo Lagoon in Lake Magadi at different times of day.** Data are mean±s.e.m. (*n*=6 pools; 10 larvae per pool). Different letters indicate significantly different mean values among chironomids sampled at different times of day for each parameter analyzed (one-way analysis of variance followed by Tukey's test; *P*<0.05).

Activities of SOD (Fig. 5), GPx and GR (Fig. 6), as well as concentrations of reduced (GSH) and oxidized (GSSG) glutathione (Fig. 7) were significantly higher in chironomid larvae collected at 13:00 h than in those collected at other times of day. The GSH/GSSH ratio was between 19.5 and 25.3 at the different times of day, with reduced GSH corresponding to 94.8-96.0% of the total GSH content; although no significant diel variation was observed in CAT (Fig. 5) and GCL (Fig. 6) activity.

## DISCUSSION

The larval chironomid collected at the Flamingo Lagoon on the east side of Lake Magadi was identified as the larvae of *Tanytarsus minutipalpus* (Insecta, Diptera, Chironomidae). *Tanytarsus* is described as one of the most diverse genera of the Chironomidae family (Pinder and Reiss, 1983; Rasmussen, 1996; Chapman, 1998; Jurenka, 2015). *T. minutipalpus* was first described in 1999, with the occurrence of adult and larvae being reported in saline lakes of Ethiopia, Tanzania and Kenya; however, no reference was made for its occurrence in Lake Magadi in Kenya (Ekrem and Harrison, 1999). Therefore, to the best of our knowledge, this study is the first to report the occurrence of the chironomid *T. minutipalpus* in Lake Magadi.

The present study is also the first to report biochemical and physiological adjustments of an invertebrate species to natural variations in the extreme conditions of Lake Magadi. As we hypothesized, *T. minutipalpus* larvae displayed biochemical and physiological adjustments correlated with these changing conditions. As reported by Verschuren (1997), *Tanytarsus* sp. is anoxia intolerant and the only genus of chironomid to thrive in meta-saline African soda lakes. The diel variations in water temperature (20.2-29.3°C) and dissolved oxygen (3.2-18.6 mg O_2_ l^−1^) content, as well as the more or less constant water pH (10.0±0.03) found in the present study are quite similar to those previously reported for Lake Magadi (Reite et al., 1974; Johansen et al., 1975; Narahara et al., 1996; Wilson et al., 2004; Johannsson et al., 2014). As hypothesized, these variations correlated with a significantly enhanced metabolism (increased activity of cholinesterase and concentrations of glucose, cholesterol, urea, creatinine and hemoglobin) paralleled by positive and cooperative adjustments in the major components of the antioxidant system (increased SOD, GR and GPx activity and GSH concentration) in *T. minutipalpus* larvae during the natural increase in water temperature and dissolved O_2_ content. Therefore, as discussed in detail below, our findings support the idea that the observed changes are key adjustments performed by this aquatic animal in association with the diel variations in physicochemical conditions of Lake Magadi.

Cholinesterase exhibits widespread distribution in chironomid tissues, especially in the central nervous system, playing an essential role in the regulation of functional activities, including ventilation (Booth and Lee, 1971; Pitman, 1971; Toutant, 1989; Chapman, 1998; Azevedo-Pereira et al., 2011; Jurenka, 2015). Therefore, the increased cholinesterase activity observed in chironomid larvae collected at 13:00 h suggests a possible enhanced ventilation activity of *T. minutipalpus* larvae in order to support an increased rate of O_2_ consumption. Therefore, in spite of the observed higher water O_2_ content, chironomid larvae would still need to increase their ventilation. As further discussed below, this could be associated, at least in part, with increased feeding activity. However, cholinesterase activity in larvae collected at 19:00 h was similar to that observed in larvae collected at 07:00 h of the same day, as well as 01:00 h and 07:00 h of the next day. Mean water temperature and dissolved oxygen content at 19:00 h were as high as those observed at 13:00 h of the same day. Consequently, while natural increases in temperature and/or dissolved oxygen content may be triggering the observed changes in *T. minutipalpus* larvae, it is also possible that biochemical and physiological adjustments are initiated and/or terminated in response to signaling factors other than temperature. One possible explanation would be the existence of an intrinsic circadian rhythm in the chironomid larvae associated with daylight. Therefore, further studies are needed to understand the mechanisms involved in the signaling process. In summary, data reported in the present study are correlated with time and natural variations in environmental conditions, but correlation does not necessarily imply causation. Future experiments under controlled laboratory conditions designed to test the isolated and combined effects of increasing water temperature and dissolved oxygen content will be required to unambiguously link the cellular responses observed in the chironomid larvae to these specific environmental stressors.

In addition to the increased nervous cholinergic activity observed in *T. minutipalpus* larvae collected at 13:00 h, there also appeared to be enhanced metabolism in other tissues, as indicated by the significantly higher whole-body concentration of creatinine observed in larvae collected at this time. This suggests enhanced muscular activity in these larvae. In insects, the metabolism of creatine and creatinine is similar to that observed in vertebrates, and these compounds can be found in the fat body, circulating fluids and excreta of insects (Lazar and Mohamed, 1988a,b). Indeed, the level of creatine and creatinine in blood and fat body is indicative of the rate of mobilization and deposition of proteins throughout the larval development of insects (Shinzato et al., 1997).

In light of the discussion above, it seems that both nervous and muscular activities were increased in chironomid larvae collected at 13:00 h compared to those collected at other times of day. A higher whole-body activity would imply enhanced oxygen consumption. Indeed, a significantly higher concentration of hemoglobin was observed in chironomid larvae collected at 13:00 h (Table 1). Bilirubin, a metabolite from hemoglobin degradation, can be stored in the fat body of insects (Rockstein, 1978; Morgan, 2010). Therefore, the increase in hemoglobin concentration, together with the lack of change in bilirubin concentration observed in larvae collected at 13:00 h, suggest that the carrying capacity of blood O_2_ of these larvae was enhanced with respect to those collected at other times of day. Indeed, an increased rate of oxygen consumption to support larval development and growth at increasing temperatures probably occurred. In fact, hemoglobin is known to have a comparatively high affinity for O_2_ and to be widely distributed in tissues of chironomids, including those of the genus *Tanytarsus* (Lankester, 1867; Walshe, 1947; Panis et al., 1995; Rasmussen, 1996). The increased levels of dissolved oxygen content observed in Lake Magadi waters between 13:00 h and 19:00 h would enhance the O_2_ gradient between the environment and hemoglobin in the larvae, thus ensuring the diffusion of adequate amounts of O_2_ into the chironomid larvae. As for other metabolic parameters, the increased concentration of hemoglobin observed in larvae collected at 13:00 h was probably transient. Also, a transient increase in bilirubin concentration is expected to occur in larvae collected at some sampling point after 13:00 h and before 07:00 h of the following day. Unfortunately, hemoglobin and bilirubin concentrations were not measured in larvae collected within this time period.

In terms of energy budget, increased concentrations of glucose, urea, and cholesterol in larvae collected at 13:00 h suggest that the increased metabolism at this time relies on the absorption, mobilization and use of all major biomolecules (carbohydrates, lipids and proteins). The increased whole-body glucose could be explained as a typical response to changes in environmental conditions (Chapman, 1998; Jurenka, 2015), with this carbohydrate serving as the main source of energy during increasing temperature (from 07:00 h to 13:00 h). In turn, the increased whole-body urea concentration in parallel with a lack of change in total protein concentration could indicate a higher rate of protein metabolism, which is usually observed during development and growth of insect larvae (Régnière et al., 2012; Jurenka, 2015). Notably, whole-body urea concentration remained elevated for a longer period of time (from 13:00 h to 19:00 h) than the whole-body glucose level. This may mean that the first part of the increased metabolism when water temperature and dissolved oxygen levels were at maximum values during the daytime is related to food acquisition. This process requires an enhanced larval activity, but results in increased amounts of resources for growth. Indeed, the prolonged urea signal likely indicates that development and growth is occurring into the latter part of the day and evening, slowing as resources decline between 19:00 h and 07:00 h.

In insects, sterols are involved in critical functions such as components of cellular membranes, precursors for many hormones and regulators of genes involved in development processes (Canavoso et al., 2001; Behmer and Nes, 2003). Insects, differently from most other animals, have a dietary requirement for sterols because they lack the ability to synthesize sterols (Behmer and Nes, 2003). Therefore, the observed natural increase in whole-body cholesterol concentration would likely have been associated with a higher ingestion of cholesterol. In turn, a likely higher food ingestion rate in chironomid larvae collected at 13:00 h could be related to higher food availability at this time of day, associated with the natural increase in water temperature (Coe, 1966; Javor, 1989; Johannsson et al., 2014). Among the organisms already described as being part of the food web in Lake Magadi, the most likely source of food for *T. minutipalpus* larvae would be the cyanobacteria *Arthrospira* sp. (Coe, 1966; Javor, 1989; Johannsson et al., 2014).

As discussed above, a likely higher gross metabolism may be occurring in chironomid larvae collected at 13:00 h. This would imply increased food acquisition, as well as enhanced cellular production of ATP. However, the cellular use of oxygen as a final electron acceptor, as well as the consumption of reduced coenzymes (NADH and FADH_2_), produced in the aerobic pathways at the electron transport chain, generates a variety of ROS. It can be assumed that 0.1% of the utilized oxygen consumed is converted into free radicals (Bachschmid et al., 2013). However, higher rates of ROS generation can be observed during increasing aerobic metabolism associated with increases in water temperature (Fridovich, 2004). Although there was a lack of change in ROS concentration in chironomid larvae collected at 13:00 h, the increased SOD activity observed in these larvae is evidence of increased ROS generation. This is supported by the fact that superoxide is the first free radical generated during oxidative processes such as the electron transport chain and the xanthine/xanthine oxidase system. The superoxide radicals undergo dismutation and are converted into hydrogen peroxide in a reaction catalyzed by SOD. Indeed, this enzyme is considered as the first line of defense against the superoxide radical (Fridovich, 1997). Therefore, the higher SOD activity observed in chironomid larvae collected at 13:00 h in comparison to those collected at other times of day suggests that these larvae are producing more superoxide radicals during the period of increasing temperature and dissolved oxygen content (from 07:00 h to 13:00 h) in Lake Magadi.

The superoxide radical generated by oxidative processes can act as either an oxidizing or a reducing agent, thus generating other ROS which are more aggressive toward biomolecules (Fridovich, 1995). This free radical can induce the generation of the hydroxyl radicals through the Fenton and Haber-Weiss reactions, as well as react with hydroxyl radical generating the singlet oxygen (Sies, 1985). In fact, hydroxyl is one of the most reactive free radicals, interacting with and oxidizing all kinds of major biomolecules (Halliwell and Gutteridge, 2007).

The hydrogen peroxide produced after dismutation of the superoxide radical could be converted into water in a reaction catalyzed by CAT or GPx (Hermes-Lima, 2004). No significant diel variation was observed in CAT activity in the larvae of *T. minutipalpus*. Neither significant diel change in TOSC, nor any indication of altered levels of oxidative damage [LPO and number of apurinic/apurymidinic (AP) sites] was observed in chironomid larvae. These findings support the importance of the increased GPx activity observed in chironomid larvae collected at 13:00 h. Indeed, GPx is a major enzyme involved in the degradation of hydrogen peroxide and elimination of organic hydroperoxides (LOOH) (Fridovich, 1997; Hermes-Lima, 2004). Therefore, our data suggest that that GPx may play a key role in the antioxidant system of *T. minutipalpus* larvae from Lake Magadi. They also suggest that CAT seems not to be involved in the adjustments of the antioxidant system of *T. minutipalpus* larvae during the diel variations in water temperature and dissolved oxygen content naturally observed in Lake Magadi. An alternative explanation for the lack of diel changes in CAT activity is that this enzyme could be continuously working at its maximum activity. In this case, CAT regulation would not follow a diel pattern, unlike SOD, GPx and GR.

Reactions catalyzed by GPx have GSH as the reducing agent (Meister, 1988). Therefore, an expected higher generation of ROS would lead to a higher rate of GSSG production. In fact, a higher whole-body GSSG concentration was observed in larvae of *T. minutipalpus* collected at 13:00 h. This finding is in complete agreement with the increased SOD activity observed in these larvae. After the formation of GSSG by oxidative processes, the cellular level of GSH is restored through the oxidizing-reducing cycling mediated by the GR-GPx enzymatic system (Fridovich, 1997; Hermes-Lima, 2004). The proportional and significant increase in GPx and GR activities observed in larvae collected at 13:00 h is strong evidence that these two enzymes are working cooperatively, making the GSH/GSSG cycling a pivotal mechanism in the antioxidant system of larval *T. minutipalpus*.

In addition to the cycling system involving GPx and GR, cellular levels of GSH can be increased after a *de novo* synthesis of GSH mediated by two ATP-dependent enzymes, glutamate-cysteine ligase (GCL) and glutathione synthase (GS). GCL catalyzes the reaction between glutamate (Glu) and cysteine (Cys) to form the dipeptide γGlu-Cys, while GS combines the dipeptide with glycine to generate GSH (Maher, 2005). In the present study, we found no significant difference in GCL activity in the whole-body of *T. minutipalpus* larvae collected at 07:00 h and 13:00 h. This finding suggests that *T. minutipalpus* larvae were able to scavenge ROS produced without changing the rate of GSH production, thus reinforcing the major role of the GPx-GR system in the maintenance of the oxidative status in the chironomid larvae. However, the involvement of other non-enzymatic antioxidants cannot be ruled out – e.g. metallothioneins, biliverdin and mucoproteins (Monserrat et al., 2007). In the present study, we measured the whole-body concentration of mucoproteins in *T. minutipalpus* larvae collected at 07:00 h and 13:00 h, however, there was no significant difference among the two groups of chironomids. This finding reinforces the hypothesis that GSH plays a major role in scavenging both superoxide and hydroxyl radicals generated during the oxidative metabolism in *T. minutipalpus* larvae. In fact, we found that the oxidized form (GSSG) varied between 4.0 and 5.1% of the whole-body GSH content in *T. minutipalpus* larvae over a 24-h period. This finding illustrates the great potential of this antioxidant when the chironomidae larvae are exposed to the natural diel changes in the environmentally stressful conditions of Lake Magadi. Interestingly, the percentage of GSSG also varied within a narrow range (3-5% of the total GSH pool) in the goldenrod gall former, *Eurosta solidaginis* (Diptera: Tephritidae), during winter acclimatization (Denlinger and Lee, 2010).

In addition to superoxide and hydroxyl radicals, other free radicals such as peroxyl and alkoxyl can be also generated from organic hydroperoxides and oxygen-centered radicals, respectively. It is known that these ROS can act as intermediaries in LPO, and other oxidizing processes (Bergendi et al., 1999). As no significant diel variation in oxidative damage (LPO and AP sites) was observed, we can suggest that no significant amounts of peroxyl and/or alkoxyl were generated.

As a higher concentration of creatinine was observed in chironomid larvae collected at 13:00 h, we can infer that these larvae were more active at this time of day. In this case, an increased body (muscular) activity could lead to a higher production of reactive nitrogen species, as observed during exercise in humans (Souza Júnior et al., 2012). The nitric oxide system is also well characterized in insects (Müller, 1997). Therefore, as observed for oxygen-centered free radicals, cellular oxidizing processes can also lead to the generation of nitrogen-centered free radicals. Although NO is a gas, which plays important biochemical and physiological roles, it can also be considered as a free radical. This reactive nitrogen species (RNS) is generated through the activity of nitric oxide synthase, an enzyme involved in cellular signaling and immunological responses. In oxidative metabolism, NO is associated with the generation of the peroxynitrite radical (ONOO^−^) (Dusse et al., 2003); however, as observed for ROS, no significant variation in whole-body NO concentration was seen in *T. minutipalpus* larvae collected at different times of day. This finding suggests that RNS are not involved in the adjustments performed by the antioxidant system of *T. minutipalpus* larvae in association with the natural diel variations occurring in physicochemical parameters of Lake Magadi waters.

As discussed above, both oxygen- and nitrogen-centered radicals can lead to an oxidative stress condition with consequent damage to biomolecules. The most common damages are LPO, protein carbonylation and DNA oxidation (Dusse et al., 2003; Giorgio et al., 2007). Peroxidation of membrane lipids is one of the main causes of cell lesions and death (Abuja and Albertini, 2001). However, an organism undergoes oxidative stress only when the concentration of ROS, and likely RNS, overwhelms the organism's antioxidant capacity. Data reported in the present study clearly show that larvae of *T. minutipalpus* did not undergo an oxidative stress condition associated with the natural diel variations in temperature and dissolved oxygen content observed in Lake Magadi. This is supported by the fact that no significant changes in the levels of damage to lipids and DNA were observed, in accordance with the lack of significant changes in TOSC level, as well as ROS and NO concentrations, in chironomid larvae collected at different times of day. Therefore, it is clear that larvae of *T. minutipalpus* have an efficient antioxidant system to scavenge all of the pro-oxidants generated when facing the natural changes in the stressful conditions of Lake Magadi. Interestingly, the antioxidant system also served to counteract the ROS production in adult beetles, *Alphitobius diaperinus* (Coleoptera: Tenebrionidae), during exposure to severe cold conditions (Lalouette et al., 2011). A similar finding was also reported for the goldenrod gall former, *E. solidaginis* (Diptera: Tephritidae), a freeze-tolerant insect adapted to harsh environments (Martel, 1995). In addition to the efficiency of the antioxidant system in ROS scavenging discussed above, the involvement of other mechanisms to prevent ROS formation and/or repair of molecules affected by ROS (Storey, 1996; Ribeiro et al., 2005) cannot be disregarded. Notably, efficient protection provided by the antioxidant system against hydroxyl radicals relied on the important GSH pool in the chironomid *T. minutipalpus*, as well as in the beetle *A. diaperinus* (Lalouette et al., 2011) and the fly *E. solidaginis* (Martel, 1995).

In summary, in the present study we analyzed a large suite of biomarkers related to metabolism and the antioxidant system involved in ROS scavenging in the larvae of the chironomid *T. minutipalpus* from Lake Magadi. Considering that major enzymes involved in this scavenging capacity are SOD, CAT, GPx and GR (Michiels et al., 1994), our findings clearly indicate that all enzymatic components of a typical antioxidant system are present in the chironomid larvae. However, the mechanism involved in ROS scavenging in *T. minutipalpus* larvae comprises not only antioxidant enzymes, but also ROS-binding (non-enzymatic) antioxidants. GSH was shown to be abundant and play a crucial role in the antioxidant system of *T. minutipalpus* larvae. Indeed, GSH concentrations were found to be on average 20-fold higher than those of GSSG at any time of day. This is indicative of the importance of GSH as a non-enzymatic antioxidant for the whole-body antioxidant capacity of the chironomid larvae. This role is even more important when we consider the following facts: hydroxyl radicals are highly toxic to biomolecules; there are no known enzymatic defenses against these free radicals (Bains and Shaw, 1997); and GSH is particularly efficient in protecting against the adverse effects of hydroxyl radicals. Therefore, our findings support the idea that larvae of the chironomid *T. minutipalpus* rely on a very important pool of GSH to avoid potential oxidative stress imposed by the natural diel changes observed in the extreme and harsh physicochemical conditions of Lake Magadi waters. In the chironomid *T. minutipalpus*, our findings clearly show that this GSH pool is kept at adequate levels by an active and efficient oxidizing-reducing cycling system mediated by GPx and GR.

## MATERIALS AND METHODS

All experiments complied with the laws of Kenya, and were performed under a research permit (No. NCST/RRI/12/1/MAS/99) issued by the National Council for Science and Technology of the Ministry of Higher Education, Science, and Technology of the Republic of Kenya.

### Chironomid sampling and water physicochemical analysis

Chironomid larvae were collected from 29 July to 02 August 2010 and from 20 to 29 July 2013 at Flamingo Lagoon, which is one the four ponds or lagoons belonging to the Fish Springs Lagoon complex (1.867°S, 36.267°E) located on the east side of Lake Magadi (Magadi Township, Kajiado County, Rift Valley, Kenya, Africa) (for maps and details see: Johannsson et al., 2014). On different days of the two years of field expeditions, chironomid larvae were collected at different times of day (07:00 h, 13:00 h and 19:00 h of the first day; and 01:00 h and 07:00 h of the following day). These sampling time points were selected to cover a complete and continuous 24-h sampling period, allowing us to evaluate changes in biochemical and physiological parameters associated with the natural diel variations in extreme environmental conditions in Lake Magadi. Collections were performed at the lake shore (<0.7 m depth) using a beam trawl net (90 µm mesh). During larvae collection, the pH, dissolved oxygen content and temperature of surface water were measured (model DMO-2, Digimed, São Paulo, SP, Brazil). Based on results from the 2010 expedition (see Results section), larval sampling in the 2013 expedition was only performed at 07:00 h and 13:00 h of the first day (see Results section). In fact, increases in water temperature and dissolved oxygen content were always observed between these times of day (Johannsson et al., 2014).

Immediately after collection, larvae were manually separated using a plastic disposable Pasteur-type pipette, pooled (5-10 larva per pool; 4-6 pools per sampling time), frozen in liquid nitrogen and transferred to the laboratory for further analysis. In the 2010 expedition, all analyses were performed in an outdoor laboratory set up on the balcony of a house kindly provided by the Magadi Soda Company (Tata Chemicals Magadi) in the nearby Magadi Township. Some larvae were also preserved and transferred to the Department of Zoology and Ecology of the Federal University of Santa Catarina (Florianópolis, Santa Catarina State, Brazil) for taxonomical identification. In the 2013 expedition, all analyses were performed in an indoor laboratory set up at the Magadi Secondary School (Magadi Township). Additionally, live larvae were transferred to this laboratory in water from the collection site for ROS concentration analysis.

### Sample preparation, reagents and biochemical analyses

In the laboratory, frozen samples were weighed (wet weight) using an electronic scale (model AD500S, Marte Científica, São Paulo City, São Paulo State, Brazil), homogenized in cold saline solution (0.9 g l^−1^ NaCl) using an ultrasonic processor (Sonaer Ultrasonics, Farmingdale, NY, USA), and centrifuged at 2800 ***g*** for 5 min (model NT800, Nova Técnica, Piracicaba, SP, Brazil). The supernatant was collected and immediately used for biochemical analyses using colorimetric methods, as described below.

The following parameters were measured using commercial reagent kits from Labtest Diagnóstica (Lagoa Santa, Minas Gerais State, Brazil): cholinesterase activity (Colinesterase Liquiform kit) and glucose (Glucose PAP Liquiform kit), triglycerides (Triglicérides Liquiform kit), cholesterol (Colesterol Liquiform kit), proteins (Sensiprot kit), urea (Uréia CE kit), creatinine (Creatinina K kit), hemoglobin (Hemoglobina kit), bilirubin (Bilirrubina kit), and mucoprotein (Mucoproteínas kit) concentration.

The following parameters were measured using reagent kits from PromoCell GmbH (Heidelberg, Germany): superoxide dismutase (SOD) activity (SOD Assay Kit), glutathione reductase (GR) activity (Colorimetric Glutathione Reductase Activity Kit), reduced glutathione (GSH) concentration (Colorimetric Glutathione Detection Kit), and DNA damage (Genomic DNA Isolation Kit I and DNA Damage Detection Kit).

The following parameters were measured using commercial reagent kits from Oxford Biomedical Research (Oxford, Michigan, USA): catalase (CAT) activity (Spectrophotometric Assay for Catalase), glutathione peroxidase (GPx) activity (Colorimetric Assay for Cellular Glutathione Peroxidase), oxidized glutathione (GSSG) concentration (Microplate Assay for GSH/GSSG), glutamate-cysteine ligase (GCL) activity (GCLM Elisa kit), nitric oxide (NO) concentration (Nitric Oxide Colorimetric Assay Kit), total oxyradical scavenging capacity (TOSC) level (Total Antioxidant Power Kit), and lipid peroxidation (LPO) level (Lipid Peroxidation Microplate Assay Kit).

In all cases, we followed the instructions of the reagent kit manufacturer. Whenever possible, assays were adapted to a 96-well microplate. Therefore, sample absorbance readings were performed using either a microplate reader (model ELx-800, BioTek, Winooski, VT, USA) or a spectrophotometer (model Spectronic 20, Milton Roy Company, Houston, TX, USA). Data were expressed considering the wet body weight or the protein content in the larval homogenate.

Upon arrival in the laboratory, live larvae were immediately used for ROS measurements. They were pooled, homogenized and centrifuged as described above for frozen samples. However, samples were homogenized using a phosphate-buffered saline solution containing NaCl (140 mM), potassium phosphate buffer (10 mM, pH 7.0) and dextrose (5.5 mM). ROS concentration was determined in the homogenate supernatant using the colorimetric method described by Fowler et al. (2003), which is based on the measurement of H_2_O_2_ using phenol red and peroxidase. A standard curve was developed with solutions of H_2_O_2_ at different concentrations (7.5-30 µM) prepared in the homogenizing buffer. Protein content in the homogenate supernatant was measured as described above for the frozen samples. Data were expressed as µmol H_2_O_2_ mg protein^−1^.

### Data presentation and statistical analysis

Data were expressed as mean±standard error (*n*=6 pools of 10 larvae per pool). Data were first tested for homogeneity of variance using the Cochran’s C test. In turn, data normality was checked by the normal probability plot of raw residuals. Data for all parameters were parametric (normal distribution and homogeneous variance). Therefore, mean values were compared using one-way analysis of variance (ANOVA) followed by Tukey's test. In all cases, the significance level adopted was 95% (α=0.05).
